# Spent fowl as a source of unintentional egg proteins exposure in Canadian food products

**DOI:** 10.1016/j.psj.2022.102003

**Published:** 2022-06-11

**Authors:** Jérémie Théolier, Gabrielle Vatin, Virginie Barrère, Silvia Dominguez, Samuel Benrejeb Godefroy

**Affiliations:** Food Risk Analysis and Regulatory Excellence Platform, Institute of Nutrition and Functional Foods, Laval University, Québec, Canada

**Keywords:** spent fowl, egg, allergen, poultry processing, ELISA

## Abstract

The occurrence of egg proteins in products containing spent fowl manufactured under current practices was studied to assess the risk these food products may pose to egg-allergic consumers and to determine if Precautionary Allergen Labelling (**PAL**) was recommended. Spent fowl slaughtering and processing operations in 2 Canadian facilities were observed. Raw hen pieces (n = 134), coming from 2 facilities, and intermediate and processed products containing spent fowl (n = 57), coming from one facility, were analyzed using ELISA. All samples tested positive for egg proteins. Raw pieces were tested using a qualitative method (i.e., swabbing); estimated egg proteins concentrations suggest the presence of highly contaminated samples (>600 mg/kg in 2 hen wing samples). Swabbing was found to be efficient for rapid detection of eggs in raw hen pieces, but not for quantification. A comparison between swab and grind results showed that egg proteins concentration is underestimated by at least a factor 2 for whole carcasses and a factor 10 for breast, wings and drumsticks, when using the swab protocol. For intermediate and processed products, quantitative measurements indicate that egg protein levels were below 16 mg/kg. Additionally, 88 water samples from chiller tanks were analyzed and indicate that this step could be the cause of the global contamination observed with an increase in egg protein concentrations overtime during the production schedule. As egg contamination is not adequately controlled under the current good production practices, the use of PAL would be recommended for raw spent fowl products.

## Graphical abstract

**Figure:**
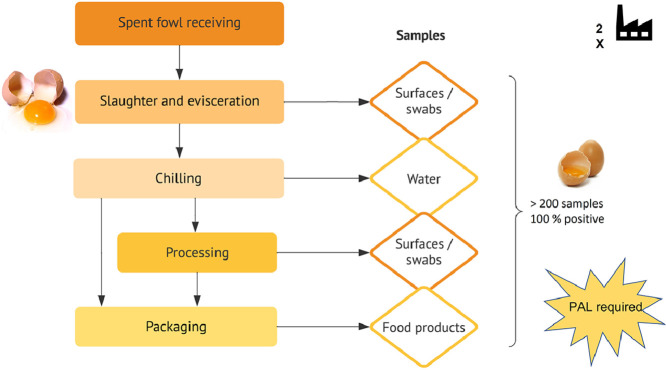

## INTRODUCTION

The occurrence of egg allergy in the Canadian population is estimated to be around 0.9% overall: 2.1% among children and 0.7% among adults (Clarke et al., 2020). About half of allergic children will acquire tolerance to eggs as they grow up (Savage et al., 2007; Dang et al., 2019; Andorf et al., 2020). Desensitization may increase the quantity of allergen tolerated (Chomyn et al., 2021), however, avoidance of the culprit ingredients remains the most effective strategy to prevent allergic reactions. When purchasing prepackaged foods, allergic consumers rely on the information on the product's label to make safe choices. Eggs are on the list of priority allergens in food in many countries (Gendel, 2012) including Canada, which means that eggs must be declared on the list of ingredients when they are intentionally added to a food product. In addition, the Safe Food for Canadians Regulations (**SFCR**) require Food Business Operators (**FBO**s) to implement preventive controls for hazards, including allergens, that present a risk of contamination of a food (Government of Canada, 2018). Nevertheless, the undeclared presence of allergens in food products is a leading cause of food recalls in Canada.

FBOs apply different strategies to control allergen hazards in their facilities, processes, and products (e.g., specification of raw material, preventive measures on site: cleaning, identification, segregation). When preventive controls cannot avoid unintentional allergens in finished products, FBOs use Precautionary Allergen Labelling (**PAL**) as a risk mitigation strategy. As a tool for FBOs, PAL is not mandatory in Canada. However, its use should be based on an evidence-based risk assessment, according to Health Canada guidelines (Government of Canada, 2012), and should be applied only when the unintentional presence of allergens in food is unavoidable, despite all reasonable measures. Still, PAL is currently widely used by FBOs which raises concerns about its reliability (DunnGalvin et al., 2015). To illustrate, a study on the occurrence of allergens in products with PAL sold in Canada (Manny et al., 2021a) observed that only 7% of products with PAL for eggs contained detectable concentrations of eggs. Spent fowls, defined as “laying hens at the end of egg production or breeders at the end of breeding production” by the Canadian chicken licensing regulations (Government of Canada, 2002), are not primarily used for their meat, but they can be used in processed products when their productivity declines. Since the reproductive system of hens is likely to contain eggs before the animals are sent to the slaughterhouses, they could represent a source of unintentional egg proteins. In this case, the need to communicate this risk, if applicable, to consumers through PAL should be investigated.

According to estimated dose response curve, a quantity of egg proteins as low as 0.2 mg can trigger an objective allergic reaction in 1% of the egg allergic population (Houben et al., 2020; Remington et al., 2020). Poultry-based food products are produced and consumed at high volumes in Canada (USDA Foreign Agricultural, 2019), and it is unclear if consumers are aware that they could be consuming products containing spent fowl and potentially egg proteins. Even if it is the case, for example, if these products include PAL for eggs, Marchisotto et al. (2017) reported that 40% of North American allergic consumers declared purchasing products with PAL. Thus, we investigated the occurrence of egg proteins in the production environment to assess the risk of cross contamination, and the occurrence of egg proteins in products containing spent fowl manufactured in Canadian facilities, to assess the risk they may pose to egg-allergic consumers and to guide risk management strategies for food manufacturers.

## MATERIALS AND METHODS

Three poultry slaughtering and processing facilities from three Canadian provinces participated in this study, conducted between January 2019 and March 2021. These facilities were identified by the Canadian Poultry and Egg Processors Council as representative of large poultry processing operations using spent fowl in Canada. Results from only 2 facilities are presented here, as the sampling protocol used in the third facility was different due to Covid-19 restrictions.

### Analytical Methods

All samples were analyzed in duplicate using sandwich-type ELISA methods. The RIDASCREENFAST Egg (R-Biopharm A.G., Darmstadt, Germany) kit was used for raw samples, and the Morinaga FASPEK Egg (Ovalbumin) (Morinaga, Tokyo, Japan) for cooked or precooked samples, as recommended in the literature (Khuda et al., 2012; Manny et al., 2021a). Method instructions provided with the kits were followed, except for swabs ELISA tests, that were done in accordance with manufacturer's guidance (R-Biopharm, 2015). Samples analyzed with Morinaga were extracted overnight. All ELISA plates were performed manually, and the optical density was measured in a spectrophotometer (ThunderBolt – Gold Standard Diagnostics, Davis, CA). Results were analyzed using the RIDASOFTWin.NET software (R-Biopharm A.G., Darmstadt, Germany) for both kits and expressed in egg proteins. Limits of quantification (**LOQ**) were 0.24 and 0.31 mg egg proteins/kg for the RIDASCREENFAST Egg and the Morinaga FASPEK Egg kits respectfully.

### Egg Proteins Recoveries with Spiked Chicken

Egg recovery was assessed with pieces of egg-free, boneless, skinless chicken broiler breast (60 ± 0.5 g). These pieces were spiked with 100, 50, 25 µg of egg proteins, with spiking solution prepared following the protocol of Manny et al. (2021a) and left to dry for 1 h. Negative controls were performed using egg-free water. Both swabbing and grinding were assessed in parallel. Samples were analyzed by ELISA using the RIDASCREENFAST Egg (R-Biopharm A.G., Darmstadt, Germany) kit as follows:

(i) Three spiked chicken pieces were ground separately. Triplicates of 1 g from each were sampled and egg protein concentration was measured. Instructions provided by the kit manufacturer for the detection of egg proteins were followed.
(ii) The whole surface of 3 spiked chicken pieces were swabbed individually using a cotton swab (Greiner Bio-One, Monroe, NCA). Swabs were analyzed according to manufacturer's guidance with an ELISA kit (R-Biopharm, 2015).

### Observation of Slaughtering and Processing Operations

Both facilities processed hens and chickens, but hens were always processed on dedicated production lines. The production shifts were composed of several lots of hens coming from different producers. Differences in hens’ size were observed from one lot to another. The number of spent fowls by production shift ranged from 10,000 to 35,000 for the 2 facilities visited.

### Sample Collection and Preparation

Environmental sample collection (surface and chiller) was facility-specific for the 3 different zones investigated: slaughtering and evisceration, chilling, and packaging. Finished product samples were collected at the end on the manufacturing process, before packaging.

#### Surfaces

Equipment and conveyors surface swab samples were collected from the evisceration, chilling and packaging areas of each facility visited. For each surface, an area of approximately 10 cm^2^ was swabbed using cotton swabs (Greiner Bio-One, Monroe, NC). For smaller pieces of equipment, the whole surface was swabbed. In each facility, sampling was conducted at different times during the production run. In the evisceration area, swabs were collected before the start and a few minutes after the beginning of production. Additional sites were swabbed during production and immediately after quick cleaning. In the packaging area, swabbing was also conducted before the start of production, and each line receiving hens was swabbed during production. The different lines were not used at the same time and not necessarily for the same product during the entire production shift. Surface swabs were stored under refrigeration until analysis.

#### Chiller Tank Water

The 2 visited facilities used different types of chiller tanks. One facility used one single tank, where samples from 3 different points (entrance, middle, and exit) were collected. No water volume was added during the production shift. The other facility used 2 tanks in parallel, but not at the same time. Fresh and uncontaminated water was added in the tanks at several points during the production schedule. Water samples were stored under refrigeration until analysis. Approximately 10 mL of water were collected per sample. Samples were analyzed within 48 h. A total of 36 chilling water samples were collected from facility A (every 30 min) and 52 from facility B (every 15 min during the first 2 h and every 30 min after this point). Each water sample was mixed, and 1 mL was used for sample preparation. ELISA kit manufacturer's instructions were followed for analysis. Additional egg-free water samples were spiked and tested to evaluate the impact of antimicrobial products on egg protein detection.

#### Products

A total of 191 food product samples were tested, including not-ready-to-eat (**NRTE**) and ready-to-eat (**RTE**) refrigerated and frozen products (Table 1). Hen and hen-based products collected on-site were sampled at different times during the production run.

**Table 1:** Description of products tested.

Product category	Number of samples	Storage conditions	Pre-cooked	Sample collection
Whole carcasses	33	Refrigerated	No	Collected on-site
Hen pieces (breasts, drumsticks, and wings)	101^a^			
MSM	9			
Frankfurters	8	Refrigerated	Yes	
Wieners	4			
Bologna	6			
Frankfurters	5	Refrigerated	Yes	Shipped by manufacturers
Wieners	5			
Cooked roast	5			
Bologna	5			
Nuggets	5	Frozen	Yes	
Burgers	5		No	
**Total**	191			

a Forty-five samples from facility A and 56 from facility B.

From facility A, 78 raw meat products (33 whole carcasses and 45 drumsticks and wings) were collected and stored under refrigeration to the university until analysis within 48 hours (Table 1). The 33 whole carcasses were collected every 5 min during a 2 h and 40 min period, at the very end of the production line, immediately before final packaging. Cotton swabs were used to swab an area of approximately 10 cm^2^ of each piece. Random samples (unboned whole carcasses) were ground after swabbing to confirm the results and establish a potential correlation between the swabbing and grinding results. The 45 hen pieces were collected at 3 separate schedule points (15 pieces at each time point) to investigate a potential evolution of the egg concentration in samples during the production shift. These pieces were entirely swabbed and analyzed individually.

From facility B, 56 raw meat products (breasts, legs, and wings) were collected and swabbed on-site. Samples were collected every 15 min during a 6-h range with 2 exceptions (8 h and 12 h after the start of the production). However, the different products were not produced continuously, and some production stops were observed on site. The protocol previously described was used for swabbing, except that for small pieces (e.g., wings, drumsticks, or breasts), the entire surface was swabbed on site and the swabs were stored under refrigeration for 48 h before analysis. In addition, 12 samples of raw meat products (grinding), 9 samples of mechanically separated meat (**MSM**), and 18 samples of processed products were analyzed. Samples were kept under refrigeration during transport and storage. This facility also shipped 30 additional samples of frozen processed products (6 products from 5 different lots) for analysis. These samples were kept frozen during shipping and were either refrigerated or frozen during storage (Table 1). For processed products composed of several small units (e.g., nuggets, wieners), composite samples of approximately 200 g were prepared from units of the same package, by grinding in a Grindomix GM200 (Retsch, Haan, Germany) to obtain a paste. Frozen product samples were defrosted overnight at 4°C before preparation and analysis. For cooked roasts (3.5 kg piece per package), each roast was cut with an egg-free knife in several pieces and approximately 200 g were ground and analyzed as an individual sample. MSM samples were not ground before analysis.

### Statistical Analysis

The means of egg proteins concentrations measured in products containing spent fowl were compared with one-way analysis of variance (**ANOVA**) followed by Tukey's HSD tests. Statistical analyses were performed using RStudio v1.2.5033 (RStudio Team, Boston, MA).

## RESULTS

### Preliminary Tests

Recovery rate using the grinding method was estimated to be 84% (±6.9) when breasts were spiked with 100 µg of egg proteins. However, results were under LOQ for the 2 other spiking solutions tested (i.e., 50 µg, 25 µg). For the swabbing procedure, recoveries were always below 5% but no negative results were observed for the three spiking solutions. Breasts spiked with egg-free solution were below LOQ and swabs with or without egg-free spiking solution were also below LOQ. Based on these results, it was decided that hens and hen pieces would be swabbed when analyzed for egg proteins detection, and ground when analyzed for egg proteins concentration. The grinding method was selected for processed products since egg contamination, if present, was expected to be homogenous and high.

### Nonfood Samples

Contamination with egg proteins on the evisceration area's equipment and conveyors surfaces, immediately after cleaning, was minimal. Out of 17 sites tested, eggs were detected in 6 (0.51–3.91 mg/10 cm^2^). In addition, the inside tip of a handheld sprayer, an equipment identified on-site as difficult to clean, was tested and found to be contaminated at levels that could not be quantified after 1:20 dilution. Rapid contamination in the evisceration area was observed and measured once operations started, increasing as much as 4 orders of magnitude (mg egg proteins/10 cm^2^) in 15 min. Egg yolk residues were clearly visible on working surfaces and conveyors after a few minutes of processing. The smallest measured increase in 15 min was from 0 to 5.7 mg egg proteins/10 cm^2^ for a bird washer, and the largest, from zero to forty 330.5 mg whole egg proteins/10 cm^2^ for a conveyor entering the chilling area. Egg proteins were detected in all conveyors transporting hens from the evisceration area to the chilling area.

**Figure 1:**
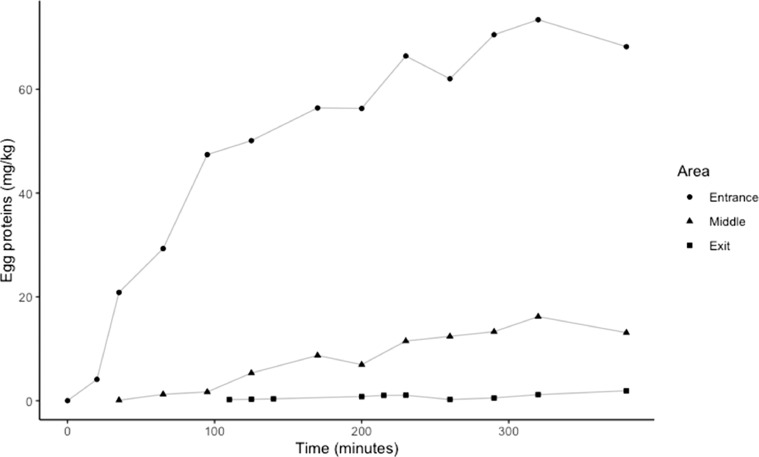
Concentration of egg proteins (mg/kg) over time in three areas of a chiller tank (Facility A).

In the facility with the unique water tank (facility A), a decreasing gradient of egg concentration was measured over time as the hens circulated from the entrance to the exit of the tanks (Figure 1). Chilling water was contaminated as early as 20 min after the immersion of the first hens and increased steadily over time at the entrance area of the chiller (maximum concentration measured = 73.4 mg egg proteins/kg). The middle and exit areas of the chiller were contaminated at a slower rate and the levels of egg proteins detected in the water were lower. The maximum concentration of egg proteins measured in the middle and exit areas of the tank were 16.2 and 1.91 mg/kg, respectively.

In facility B, the egg concentrations fluctuated over time due to the addition of fresh non contaminated water. However, once egg proteins were detected in a tank, all the following samples tested positive for egg proteins (0.1–7.56 mg/kg).

In both facilities, the surfaces of conveyors exiting the chilling areas and entering the packaging areas, which were found to be free of egg proteins before the start of production, were lightly contaminated throughout the production run and up to the entrance to the packaging area (0.1–35.8 mg egg proteins/10 cm^2^; n = 7 conveyors, 9 different time points). Similarly, the surfaces of conveyors in the packaging area were also found to be contaminated throughout the production run (2.2–99.5 mg egg proteins/10 cm^2^; n = 10 conveyors, 10 time points). It was determined that the conveyors were quickly contaminated once the first pieces of meat passed over them.

### Food Samples

**Figure 2:**
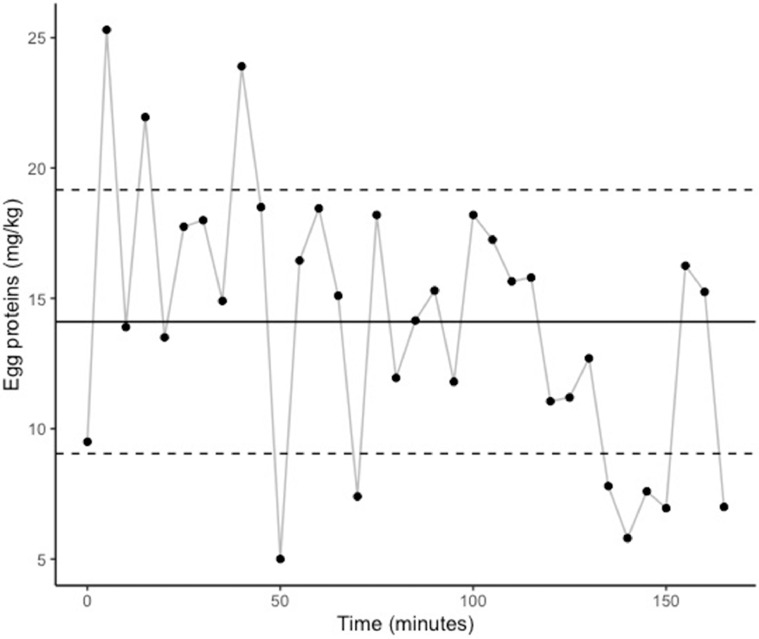
Concentration of egg proteins (mg/kg) in whole hens sampled at 15-minute intervals during packaging in facility A using swabs. Mean ± standard deviation shown in solid and dashed lines.

**Figure 3:**
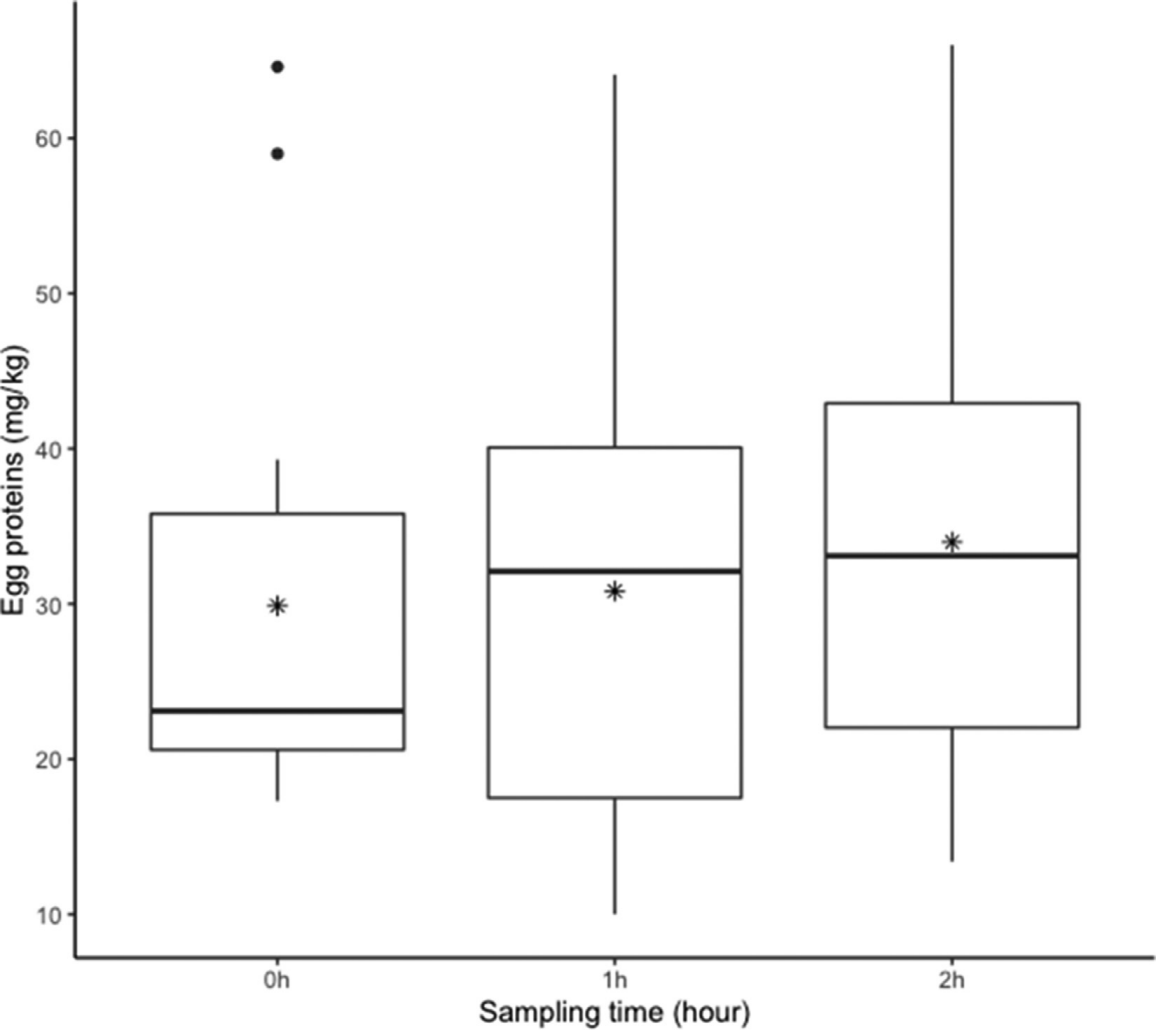
Concentration of egg proteins (mg/kg) in hen pieces sampled at 3 time points during packaging in facility A. Mean shown with (*).

A total of 191 product samples were tested by swabbing and/or grinding (Table 1). Egg proteins were detected in all 191 samples tested. In facility A, whole hen samples (n = 34) were collected from the beginning until the end of packaging at 15-min intervals. Egg protein concentrations were 14.1 ± 5.1 mg/kg when swabs were used (Figure 2), and 36.9 ± 14.3 mg/kg when samples were ground (see Table 2). The ratio between swab and grinding results was 2.2 (±0.4). In addition, hen pieces (i.e., 5 wings, 5 drumsticks and 5 breasts) were collected at 3 time points during packaging, each time point representing a composite sample of 15 different pieces that were swabbed individually and analyzed together (Figure 3). Mean egg protein concentrations in hen pieces were 13.8 ± 7.2 mg/kg at time 0, 14.3 ± 7.1 mg/kg after 1 h and 15.8 ± 7.1 mg/kg after 2 h. No statistical differences were observed among those results.

**Table 2:** Comparison between swab and grind results, expressed in egg proteins in mg/kg.

Hen piece	Sampling time	Swab result	Grind result	Ratio (Grind/Swab)*
Whole carcasses (Facility A)	11:30 PM	25.3	50.5	2.0
	11:45 PM	13.5	26.3	1.9
	0:05 AM	23.9	56.9	2.4
	0:40 AM	18.2	36.9	2.0
	1:20 AM	15.8	30.9	2.0
	2:10 AM	7.0	20.0	2.9
Breasts (Facility B)	11:00 AM	3.7	77.3	20.9
	7:45 AM	3.6	62.6	17.4
	8:45 AM	4.6	219.1	47.6
	9:30 AM	6.0	265.7	44.3
	11:00 AM	3.7	77.3	20.9
Drumsticks (Facility B)	6:00 AM	60.2	> 750	> 12.5
	7:15 AM	28.5	345.7	12.1
	8:30 AM	26.9	486.2	18.1
	8:45 AM	37.6	433.5	11.5
Wings (FacilityB)	8:00 AM	63.0	306.7	4.9
	8:45 AM	37.4	>750	>20.1
	10:45 AM	19.6	540.8	27.6

⁎ (Grind/Swab) = Egg concentration obtained with Grinding/Egg concentration obtained with swabbing.

**Figure 4:**
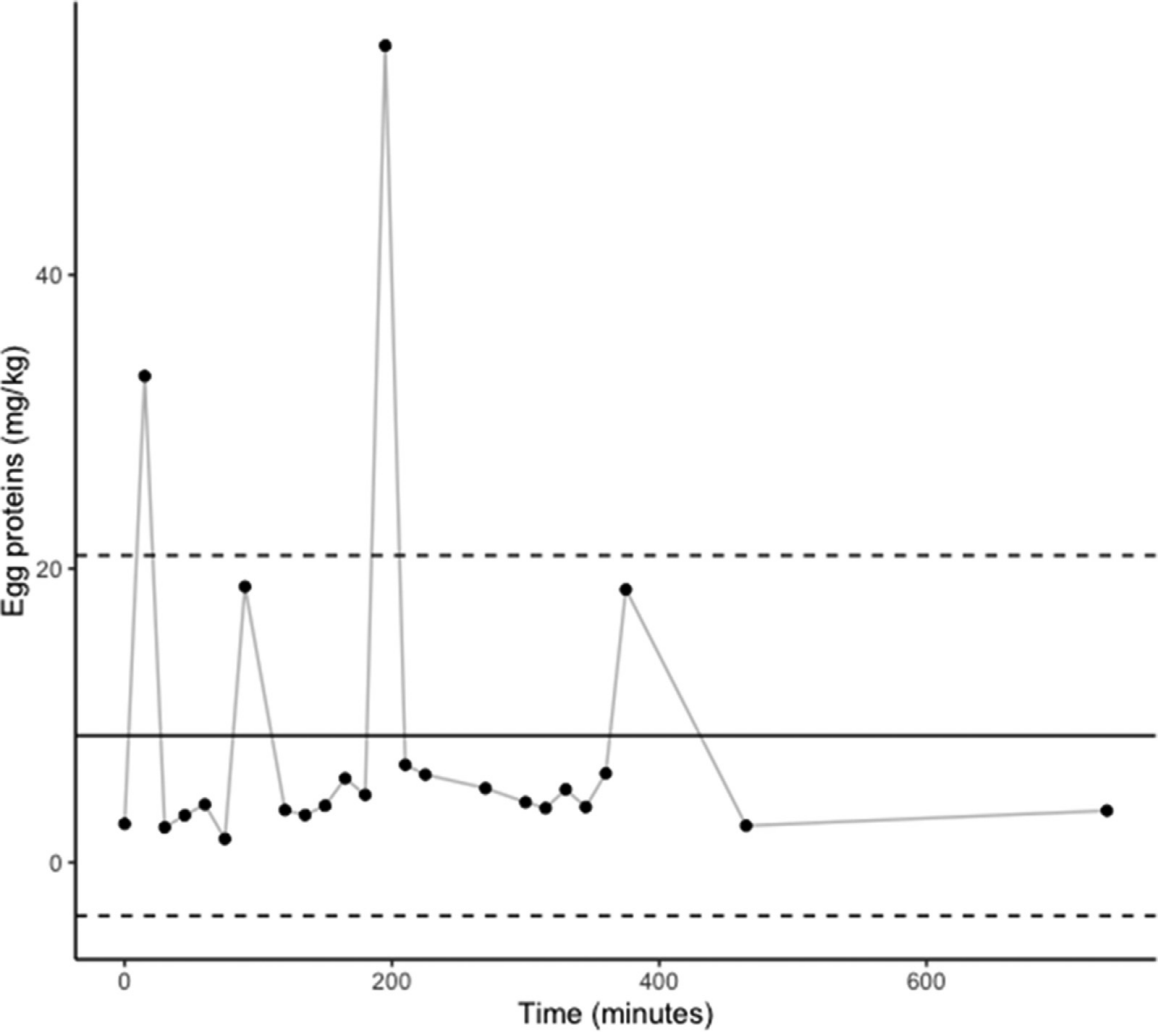
Concentration of egg proteins (mg/kg) in hen breasts sampled during packaging in facility B. Mean ± standard deviation shown in solid and dashed lines.

**Figure 5:**
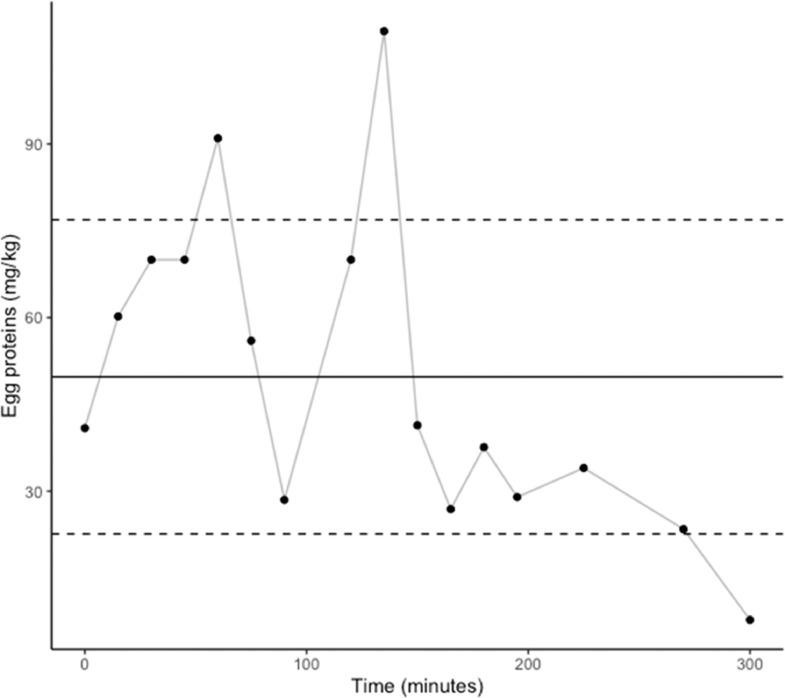
Concentration of egg proteins (mg/kg) in hen drumsticks sampled during packaging in facility B. Mean ± standard deviation shown in solid and dashed lines.

**Figure 6:**
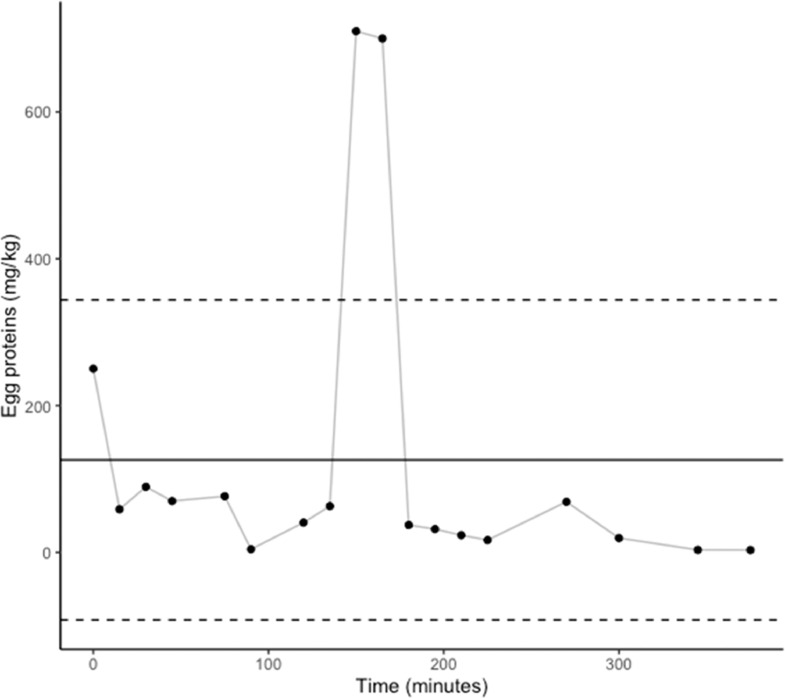
Concentration of egg proteins (mg/kg) in hen wings sampled during packaging in facility B. Mean ± standard deviation shown in solid and dashed lines.

In facility B, drumsticks, breasts and wings were studied separately during a longer time frame using the swab sampling protocol. Mean egg protein concentrations in hen breasts (n = 24) sampled during a 12-h packaging shift were 8.6 ± 12.3 mg/kg. For drumsticks (n = 16) and wings (n = 16), sampled during a 5-h period in the same shift than for breasts, detected concentrations were 49.8 ± 27.2 mg/kg and 126.0 ± 217.9 mg/kg respectively. When analyzed per piece type (i.e., breasts, drumsticks, wings), without taking time into consideration, the mean egg protein concentration for wings was significantly (P ≤ 0.01) higher than for breasts. No statistical differences were noted between drumsticks and breasts or wings. Two upper-range outliers (>20.9 mg/kg) can be noted for breasts (Figure 4), 2 (> 77 mg/kg) for drumsticks and 2 others (>344 mg/kg) for wings (Figures 5 and 6 respectively). In addition, one lower-range outlier was also observed for drumsticks. Correlation between swabbing and grinding results were not applied for these pieces due to the large variability observed (Table 2).

**Figure 7:**
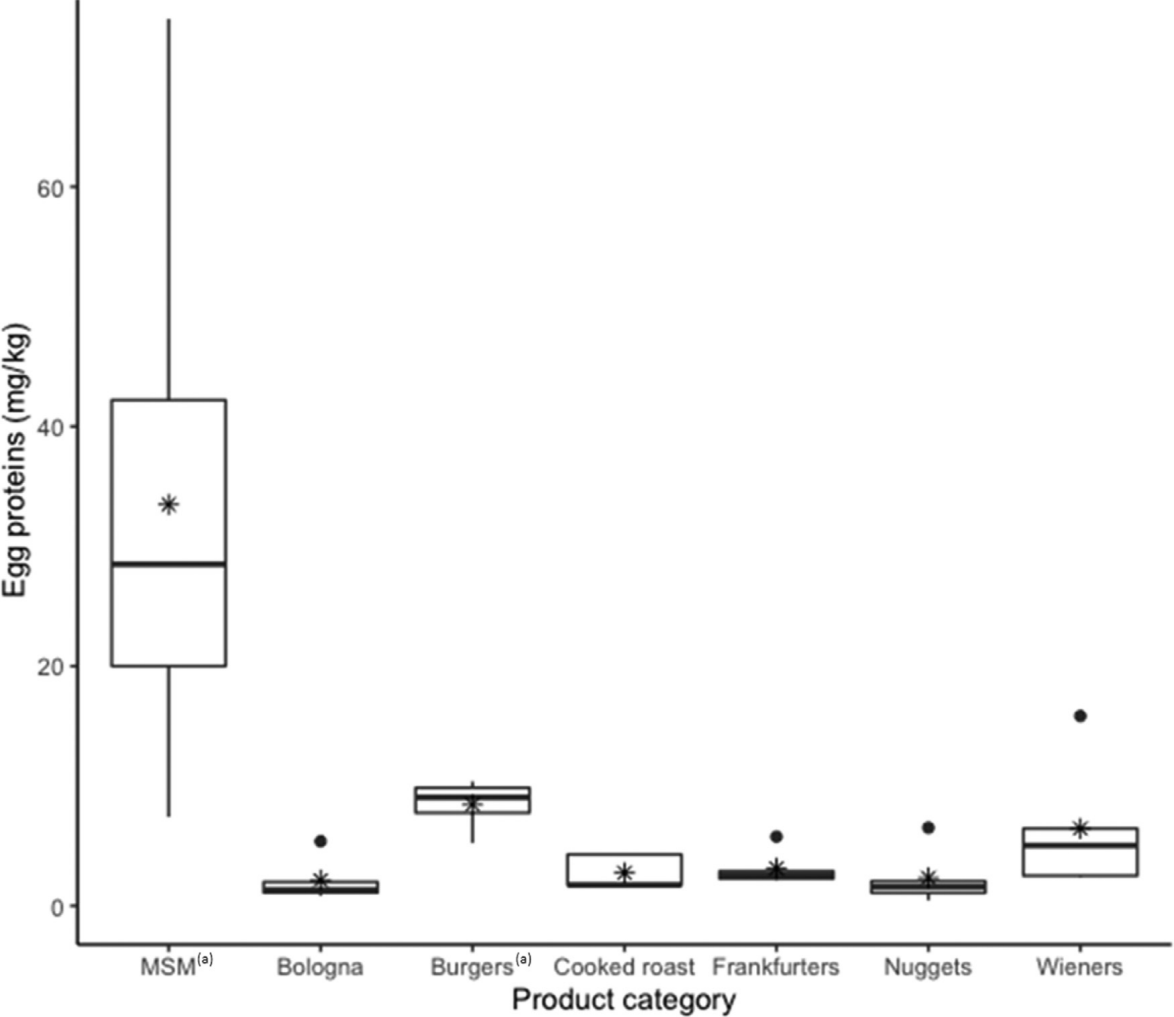
Concentration of egg proteins (mg/kg) in processed and intermediate products containing spent fowl. Mean shown with (*).^1^ Uncooked product tested with the RIDASCREEN®FAST Egg.

For the MSM samples, a mean egg protein concentration of 33.5 ± 21.3 mg/kg was measured (Figure 7). Finally, egg proteins were detected in all processed products containing spent fowl shipped by the manufacturers (n = 48) (Figure 7). The highest concentration (16.3 egg proteins mg/kg), and the highest variability (standard deviation = 6.3), were measured in wieners. Outliers, each representing one lot of products, were observed in frankfurters and nuggets. The products with the lowest mean concentration of egg proteins were nuggets and bologna (2.3 mg/kg each). Concentration of egg proteins in MSM was statistically higher (*P* ≤ 0.001) than in all processed products tested (i.e., burgers, bologna, cooked roast, frankfurters, nuggets and wieners). Among processed products (i.e., not including MSM), burgers contained significantly higher (*P* ≤ 0.05) levels of egg proteins than bologna, cooked roasts, and nuggets. All processed products tested contained PAL for eggs of the form “may contain eggs.”

## DISCUSSION

This study provides a thorough assessment of current practices in Canadian facilities processing spent fowl, focusing on the potential for egg cross-contact in finished products. Before starting the operation, the slaughterhouses’ surfaces and equipment were not contaminated with egg proteins, according to the results obtained from the 2 facilities. The cleaning-in-place and sanitation procedures were successful at removing egg proteins, even if the presence of disinfectants interferes with egg detection (reduction by a factor 3; data not shown). We believe that these negative results can be trusted. During the visits, it was readily observed that the equipment in the slaughter areas of the 2 facilities visited was contaminated with eggs once the production started. These observations were confirmed by the analytical results obtained. This contamination was caused by the mechanical separation procedures and seems inevitable in the processing environments observed. Consequently, before going in the chilling water tank, birds are likely to carry cross-contact egg proteins on their skin. Birds directed to the chilling tank may also carry eggs in their abdominal cavity (i.e., eggs at early stages of development), but these are often removed at a preceding control station. One can assume that, if there are still any egg-free carcasses at this step of the production line, they would be contaminated in the water tanks. Similarly, conveyors just after the chilling tanks were saturated with water coming from the chilling tanks via the carcasses and could also contaminate egg-free carcasses. Carcasses visibly contaminated with eggs were also occasionally observed after the chillers. According to Figure 1, egg protein tends to accumulate in chillers, without countermeasures like the addition of fresh water, which could lead to an increase in the concentration of eggs in carcasses. However, this accumulation effect could not be observed in practice on surfaces or food products. Considering these results and observations, the use of dry-air chilling may reduce the number of contaminated products, but it is mostly used for broilers and is more expensive than water chilling. According to FBOs, hen meat is not popular among Canadian consumers, which implies limited profitability, and therefore limited suitability for the use of dry-air chilling.

Initially, this project was conceived with the assumption that the occurrence of eggs in spent fowls would be low based on conversations with members of the Canadian Poultry and Egg Processors Council. These FBOs wanted to investigate if the presence of PAL for eggs was necessary in products where spent fowls were used as ingredients. Our work revealed that egg contamination in food products containing spent fowls is systemic. The concentrations of egg proteins found were also higher than anticipated. The Canadian Food Inspection Agency (**CFIA**) uses grinding as standard sample preparation method, which seems to be adequate for processed products, usually controlled by this agency. However, the use of grinders was not compatible with the sample collection protocol on-site; the use of swabs was logical given the high throughput of the production lines and the impossibility to transport hen carcasses or hen pieces in a time frame that would allow for analysis within 48 h. Yet, the swab technique is not adequate for the development of a robust risk characterization for raw poultry products, due to its qualitative nature, as observed with the preliminary tests. *De facto*, the extrapolation of swabbing results is questionable. The results obtained by comparing swab and grinding results for whole carcasses (in facility A) were consistent, probably due to the samples’ characteristics (i.e., surface larger than 10 cm^2^, and quantity of meat sufficient to obtain a 200 g bulk sample). The conversion factor (i.e., from mg egg protein/cm^2^ to mg egg protein/kg) calculated for whole carcasses did not converge with the factor determined in the preliminary tests, likely because carcasses collected from industrial settings were saturated in liquid when swabbed, whereas samples used in preliminary tests were dry. However, for smaller pieces (i.e., drumsticks, breasts, wings), correlation factors between swabbing and grinding could not be established due to high variability, but they provide a magnitude of the underestimation (at least a 10 factor). The variation of volume in small pieces – more important than in whole carcasses – likely resulted in larger variation between grinding and swabbing results and did not allow for the establishment of a correlation. It is also possible that the cotton swab used in this study is not optimal for the recovery of egg proteins, as Barrere et al. (2020) observed important differences in the recoveries of different swabs, but for milk and gliadin instead of egg proteins. According to our results, it seems that the use of swab results for extrapolation is not straightforward and would require further investigation.

In both facilities, egg cross-contact occurs early in the production shift. According to our results, this contamination seems heterogeneous, and no trend could be identified. However, one can suppose that outliers came from hens with eggs at the time of slaughter, considering that outliers were randomly spotted throughout the production shift (Figure 4, Figure 5, Figure 6). On the contrary, the other measurements would be the result of environmental contamination during evisceration and chilling. Usually, eggs are considered liquid but as there is no real homogenization step during this process, egg contamination could adhere to carcasses. Eggs can blast during the evisceration process resulting in highly contaminated spots and then chilling in water tanks seems to spread egg contamination. However, it is not sufficient to homogenize egg proteins concentration, as this is mostly a passive processing step with limited mechanical action. Consequently, cross-contact egg is found throughout the production run (i.e., homogeneous contamination) but with high concentration outliers – the latter like particulate allergens (i.e., heterogeneous contamination). This implies that the percentage of hens with eggs at the time of slaughter would be a valid criterion to assess the risk of egg contamination in products containing spent fowls. Slaughtering those hens last could reduce the presence of eggs at least in the first carcasses. In addition, higher contamination was observed in hen pieces with skin, notably wings. Since these products are more likely to contain higher concentrations of egg proteins and, considering they tend to be consumed in batches, they may pose a higher risk to egg-allergic consumers than hen breasts or whole birds. This is particularly notable because wings and drumsticks are the only spent fowl pieces consumed as such in Canada, as breasts and whole carcasses are either exported or sent to other facilities for further processing. This study demonstrates that egg contamination is not and cannot be controlled under the current good manufacturing practices and procedures investigated. Thus, even if spent fowl pieces require cooking prior to consumption, the use of PAL for eggs in these products is necessary to inform egg-allergic consumers of this risk.

Processed products analytical results revealed high occurrence and low concentration of eggs (<10 mg egg proteins/kg), except for one sample of burgers (not precooked) and wieners (high variation of contamination levels). All processed products except burgers were tested using the Morinaga kit which presents about 20% to 30% recovery rates (Manny et al., 2021a). These results appear consistent with MSM results, as expected considering these products are partially composed of MSM before further processing. Based on the nutritional portions proposed on the package, the products tested would have triggered an allergic reaction in less than 2% of the egg-allergic population, according to the eliciting doses reported by Houben et al. (2020). In addition, some of these products should require cooking prior to consumption which should further reduce the exposure to allergenic egg proteins.

Analytical results (191/191 food products tested contained egg proteins) suggest that egg contamination is not controlled, and the use of PAL should be consistent in products containing spent fowl. This is one of the few cases where the voluntary basis of PAL could be upgraded to mandatory enforcement, although not feasible in the current state of the law in Canada. To our knowledge, only a few other food products may also require this approach: dark chocolate and milk (Bedford et al., 2017; Manny et al., 2021b) and “sfouf” and sesame (Touma et al. 2021a,b). Like raw hens and hen pieces, the occurrence of cross-contact egg in processed finished products (wieners, burgers, etc.) was widespread, although at low concentrations. The use of PAL for egg in these products should be evaluated based on a robust risk assessment. However, since the current legal framework in Canada focuses on allergen presence, they could be subject to investigation and potentially, recalls. On the other hand, given the low exposure doses observed, the use of PAL based on thresholds should be considered.

## CONCLUSION

The purpose of this study was to evaluate the relevance of PAL for eggs in Raw spent fowl products (i.e., hens and hen pieces) processed under current practices in Canada. It was determined that egg contamination is systematic due the characteristics of the production process and cannot be controlled under current practices. Swabbing was found to be adequate for the rapid detection of egg in raw poultry but not for quantification. Heterogeneous egg protein concentration results were observed all along the production shifts monitored, with the presence of upper-range outliers. Therefore, according to the results of this study, the use of PAL for egg in raw spent fowl products is needed and justified. Processed finished products containing spent fowl as an ingredient (e.g., wieners, burgers, etc.), especially those sold raw or precooked, without PAL for egg, may also present a risk for egg-allergic consumers and could be exposed to food recalls, according to the current Canadian legislation. Finally, the presence of eggs in the entire production process indicates that the segregation of spent fowl-based products from other poultry products that could be considered egg-free must be required to minimize cross-contact.

## Disclosures

This work was supported through funding from the Food Regulatory Platform Fund, part of the Trust fund of Laval University, and specifically through contributions and donations provided by the Canadian Poultry and Egg Processors Council to the trust fund. The authors declare that they have no known competing financial interests or personal relationships that could have appeared to influence the work reported in this paper.

## References

[bib0001] Andorf S., Bunning B., Tupa D., Cao S., Long A.J., Borres M.P., Galli S.J., Chinthrajah R.S., Nadeau K.C. (2020). Trends in egg specific immunoglobulin levels during natural tolerance and oral immunotherapy. Allergy..

[bib0002] Barrere V., Théolier J., Lacroix S., Zbylut S., Valdez A., Collopy N., Lahey B., Godefroy S.B. (2020). Stability of milk and gliadin on swabs during 7 days under different storage conditions. Food Control..

[bib0003] Bedford B., Yu Y., Wang X., Garber E.A.E., Jackson L.S. (2017). A limited survey of dark chocolate bars obtained in the United States for undeclared milk and peanut allergens. J. Food Prot..

[bib0004] Chomyn A., Chan E.S., Yeung J., Vander Leek T.K., Williams B.A., Soller L., Abrams E.M., Mak R., Wong T. (2021). Canadian food ladders for dietary advancement in children with IgE-mediated allergy to milk and/or egg. Allergy Asthma Clin. Immunol..

[bib0005] Clarke A.E., Elliott S.J., St. Pierre Y., Soller L., La Vieille S., Ben-Shoshan M. (2020). Temporal trends in prevalence of food allergy in Canada. J. Allergy Clin. Immunol. Pract..

[bib0006] Dang T.D., Peters R.L., Koplin J.J., Dharmage S.C., Gurrin L.C., Ponsonby A.-L., Martino D.J., Neeland M., Tang M.L.K., Allen K.J. (2019). Egg allergen specific IgE diversity predicts resolution of egg allergy in the population cohort HealthNuts. Allergy..

[bib0007] DunnGalvin A., Chan C.-H., Crevel R., Grimshaw K., Poms R., Schnadt S., Taylor S.L., Turner P., Allen K.J., Baka A., Baumert J.L., Baumgartner S., Beyer K., Bucchini L., Fernández-Rivas M., Grinter K., Houben G.F., Hourihane J., Kenna F., Kruizinga A.G., Lack G., Madsen C.B., Millsa E.N.C., Papadopoulos N.G., Alldrick A., Regent L., Sherlock R., Wal J.-M., Roberts G. (2015). Precautionary allergen labelling: perspectives from key stakeholder groups. Allergy..

[bib0008] Gendel S.M. (2012). Comparison of international food allergen labeling regulations. Regul. Toxicol. Pharmacol..

[bib0012] Houben G.F., Baumert J.L., Blom W.M., Kruizinga A.G., Meima M.Y., Remington B.C., Wheeler M.W., Westerhout J., Taylor S.L. (2020). Full range of population Eliciting Dose values for 14 priority allergenic foods and recommendations for use in risk characterization. Food Chem. Toxicol..

[bib0013] Khuda S., Slate A., Pereira M., Al-Taher F., Jackson L., Diaz-Amigo C., Bigley E.C., Whitaker T., Williams K.M. (2012). Effect of processing on recovery and variability associated with immunochemical analytical methods for multiple allergens in a single matrix: sugar cookies. J. Agric. Food Chem..

[bib0014] Manny E., Vieille S.La, Barrere V., Theolier J., Godefroy S.B. (2021). Occurrence of milk and egg allergens in foodstuffs in Canada. Food Addit. Contam. Part A..

[bib0015] Manny E., Vieille S.La, Dominguez S.A., Kos G., Barrere V., Theolier J., Touma J., Godefroy S.B. (2021). Probabilistic risk assessment for milk in dark chocolate, cookies and other baked goods with PAL sold in Canada. Food Chem. Toxicol..

[bib0016] Marchisotto M.J., Harada L., Kamdar O., Smith B.M., Waserman S., Sicherer S., Allen K., Muraro A., Taylor S., Gupta R.S. (2017). Food allergen labeling and purchasing habits in the United States and Canada. J. Allergy Clin. Immunol. Pract..

[bib0011] Government of Canada. 2018. Safe Food for Canadians Regulations (SOR/2018-108). Accessed Feb. 2022. https://laws-lois.justice.gc.ca/eng/regulations/SOR-2018-108/page-10.html.

[bib0010] Government of Canada. 2012. The use of food allergen precautionary statements on prepackaged foods. Accessed Feb. 2022. https://www.canada.ca/en/health-canada/services/food-nutrition/food-labelling/allergen-labelling/use-food-allergen-precautionary-statements-prepackaged-foods.html.

[bib0009] Government of Canada. 2002. Canadian Chicken Licensing Regulations (SOR/2002-22). Accessed Feb. 2022. https://laws-lois.justice.gc.ca/eng/regulations/SOR-2002-22/FullText.html.

[bib0017] R-biopharm. 2015. RIDASCREEN® FAST – Swabbing allergen: swabbing method for the qualitative analysis of allergens in production line or for laboratory equipment.

[bib0018] Remington B.C., Westerhout J., Meima M.Y., Blom W.M., Kruizinga A.G., Wheeler M.W., Taylor S.L., Houben G.F., Baumert J.L. (2020). Updated population minimal eliciting dose distributions for use in risk assessment of 14 priority food allergens. Food Chem. Toxicol..

[bib0019] Savage J.H., Matsui E.C., Skripak J.M., Wood R.A. (2007). The natural history of egg allergy. J. Allergy Clin. Immunol..

[bib0020] Touma J., Dominguez S., La Vieille S., Remington B.C., Baumert J.L., Théolier J., Godefroy S.B. (2021). Sesame as an allergen in Lebanese food products: Occurrence, consumption and quantitative risk assessment. Food Chem. Toxicol..

[bib0021] Touma J., Vieille S.La, Guillier L., Barrere V., Manny E., Théolier J., Dominguez S., Godefroy S.B. (2021). Occurrence and risk assessment of sesame as an allergen in selected Middle Eastern foods available in Montreal, Canada. Food Addit. Contam. Part A..

[bib0022] USDA Foreign Agricultural Service. 2019. Canada Poultry and Products Annual 2019. Accessed Feb. 2022. https://www.fas.usda.gov/data/canada-poultry-and-products-annual-5.

